# Oxygen Cycling in Half-Doped Ln_1–*x*_Sr_*x*_CoO_3−δ_ Cobalt Perovskite Oxides and an In Situ Neutron Diffraction Study of Pr_0.5_Sr_0.5_CoO_3−δ_

**DOI:** 10.1021/acsomega.6c02673

**Published:** 2026-05-25

**Authors:** Fabian Hesse, Blair F. Kennedy, Emmanuelle Suard, Jan-Willem G. Bos

**Affiliations:** a Institute of Chemical Sciences, School of Engineering and Physical Sciences, 3120 Heriot-Watt University, Edinburgh EH14 4AS, U.K.; b EaStCHEM School of Chemistry, University of St. Andrews, North Haugh, St. Andrews KY16 9ST, U.K.; c Institut Laue-Langevin, 71 Avenue des Martyrs, CS20156, Grenoble Cédex 9 38042, France

## Abstract

Ln_1–*x*_Sr_*x*_CoO_3−δ_ perovskite oxides are important mixed ionic electronic conductors with applications in electrochemical energy conversion. This manuscript reports a systematic investigation of the oxygen cycling capacity of a wide range of half-doped *x* = 0.5 compositions from Ln = La–Y using thermogravimetric analysis. A similar upper limit δ_max_ = 0.35–0.40 is found, with the variation in storage capacity resulting from the highest accessible oxygen content (δ_min_). The best oxygen storage capacity of ∼0.3 mol O corresponding to ∼2 wt % is found for Ln = Pr–Sm. For Ln = Gd–Y, A-site ordered superstructures are found, linked to a deviation from the ideal 1:1 Ln:Sr stoichiometry, with compositions becoming more Sr-rich. These samples have notably reduced oxygen storage capacities. In situ neutron powder diffraction was used to probe the structural evolution of Pr_0.5_Sr_0.5_CoO_3−δ_ under flowing nitrogen gas. This revealed extensive domains of phase coexistence between *Imma*, *R*3̅*c*, and cubic perovskite structures. Significant oxygen loss occurs above 450 °C at which point the cubic phase is the largest component of the sample. This study contributes new insight into structural stability and oxygen cycling performance of these important electrode materials.

## Graphical abstract

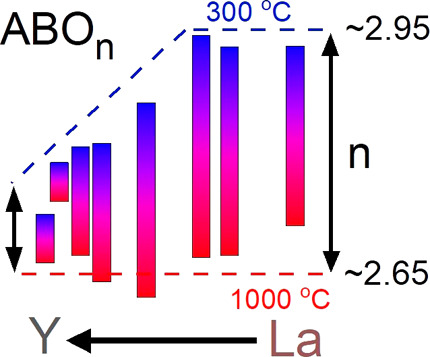

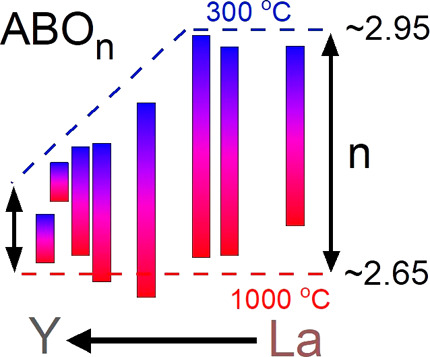

## 1 Introduction

Ln_1–*x*_A_*x*_CoO_3−δ_ (Ln = lanthanide, A = alkaline earth) perovskites are widely investigated electrode materials for solid oxide fuel cells and electrolyzers.^1–6^ These mixed electronic and ionic conducting materials have outstanding catalytic activity for the oxygen evolution and reduction. In particular, Ln_1–*x*_A_*x*_CoO_3−δ_ perovskites are able to generate large amounts of oxygen vacancies and have facile migration of bulk and surface oxygen.^1,3–5^ This work is focused on the oxygen storage capacity of *x* = 0.5, A = Sr materials for a wide range of Ln, from large La to small Y. The redox behavior of cobalt perovskites has been studied since the 1980s,^7–11^ with La_1–*x*_Sr_*x*_CoO_3−δ_ attracting by far the most attention. However, there is no comparative study of the oxygen cycling capacity over a wide range of Ln with samples prepared and analyzed in the same manner. The *x* = 0.5 composition was chosen to afford a direct comparison with the *x* = 0.5, A = Ba compositions that form a layered perovskite structure with Ln and Ba in alternating layers.^1,3^ In contrast, the *x* = 0.5, A = Sr materials have pseudocubic perovskite structures with randomly arranged A-cations.^12^ Deviations from *x* = 0.5 can lead to A-cation ordered structures as discussed below. In the Ln_0.5_Sr_0.5_CoO_3−δ_ compositions, the average charge on the A-site (2.5+) is not fully compensated by oxidizing Co^3+^ to Co^4+^, and hence, oxygen vacancies form. High temperatures and/or low oxygen partial pressures further promote the formation of oxygen vacancies.^1^

Ideal *x* = 0.5 (Ln_0.5_Sr_0.5_CoO_3−δ_) samples all have structures that are based on octahedral tilting distortions of the ideal cubic perovskite structure.^12^ An overview of the reported structures is given in Figure 1. The largest Ln = La has an *R*3̅*c* structure with a √2*a*_p_ × √2*a*_p_ × 2√3*a*_p_ unit cell, which corresponds to the a^–^a^–^a^–^ Glazer tilt structure.^13–15^ Smaller, Ln = Pr–Tb are reported with a *Pnma* structure with √2a_p_×2a_p_×√2a_p_ unit cell, corresponding to the a^+^b^–^b^–^ Glazer tilt structure. For smaller Ln, a decreasing Ln solubility is reported, yielding the highest possible Ln content Ln_0.33_Sr_0.67_CoO_3−δ_ (*x* = 0.67, Ln = Dy and Y). These compositions have an *I*4/*mmm* structure with a large 2*a*_p_ × 2*a*_p_ × 4*a*_p_ unit cell that is characterized by Ln/Sr and oxygen vacancy ordering as illustrated in Figure 1d.^16^

**Figure 1:**
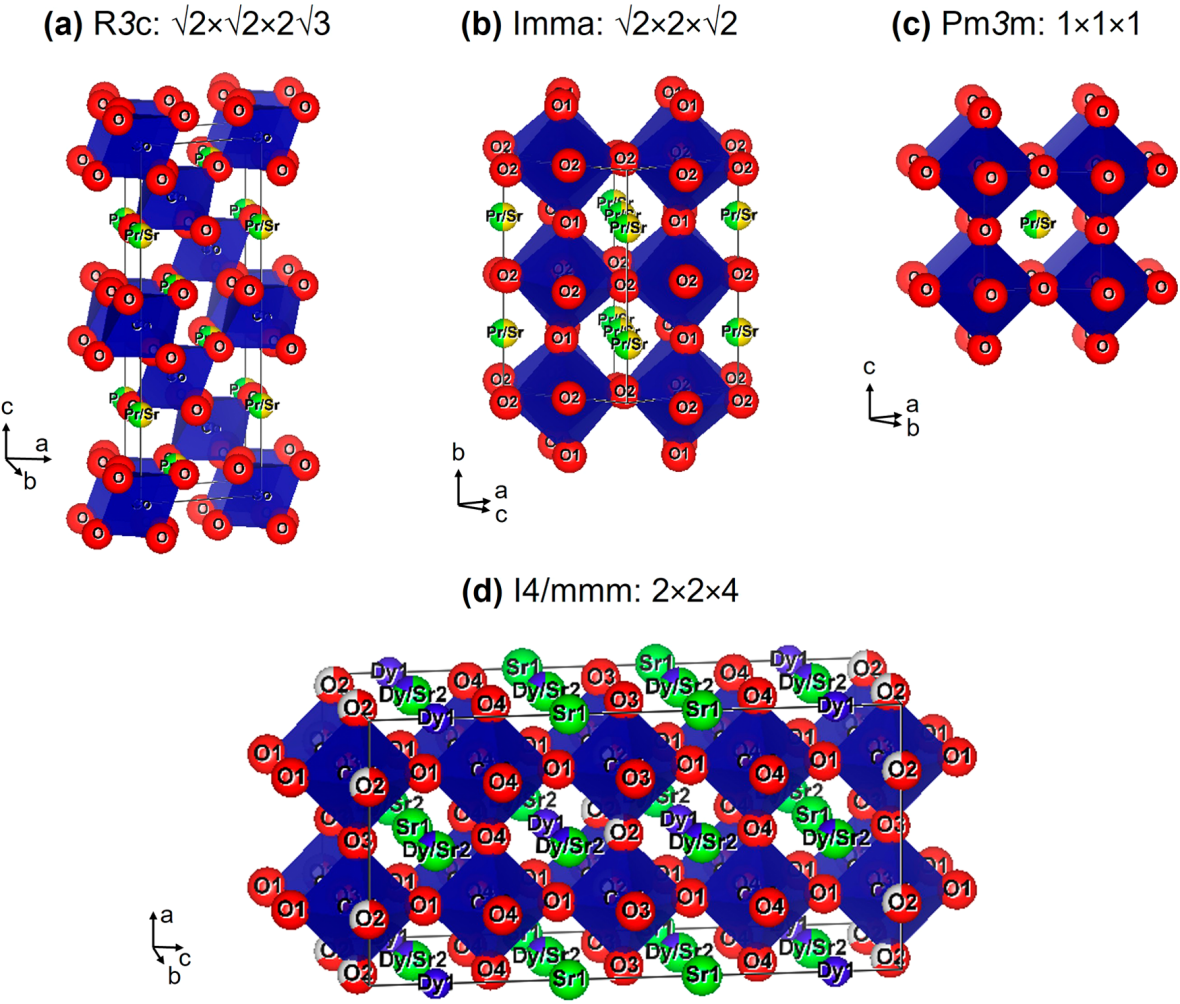
Overview of the observed perovskite structures in the Ln_1–*x*_Sr_*x*_CoO_3−δ_ materials: (a) rhombohedral Pr_0.5_Sr_0.5_CoO_3−δ_, (b) orthorhombic Pr_0.5_Sr_0.5_CoO_3−δ_, (c) cubic Pr_0.5_Sr_0.5_CoO_3−δ_, and (d) tetragonal Dy_0.3_Sr_0.7_CoO_3−δ_. Sr, Co, and O atoms are colored green, blue, and red, respectively.

Neutron powder diffraction (NPD) has been used to investigate the high-temperature structures of several of the Ln_1–*x*_A_*x*_CoO_3−δ_ (A = Sr and Ba) compositions.^17–25^ However, studies on *x* = 0.5, A = Sr compositions are relatively rare. *x* = 0.6, Ln = La was investigated in air up to 1258 °C.^26^ This revealed a structural phase transition from *R*3̅*c* to the basic perovskite structure between 400 and 600 °C and the onset of oxygen loss above 400 °C.

That study also confirmed the critical bottleneck for oxygen migration, which is a curved path restricted by a triangle of atoms made up from Co and two A cations. The *x* = 0.5, Ln = Pr composition that is studied in this work under flowing nitrogen up to 900 °C was studied previously at low temperature in inert conditions.^27^ This revealed a sequence of phase transitions from *I*4/*mcm* to *Imma* at 130 K, followed by the *R*3̅*c* structure at 320 K (47 °C). These transitions correspond to changes in octahedral tilting from *a* ^0^ *a* ^0^ *c* ^–^ → *a* ^–^ *b* ^0^ *a* ^–^ → *a* ^–^ *a* ^–^ *a* ^–^.^13^ Discontinuities are evident in the lattice parameters and cell volume at phase transition temperatures, consistent with first-order transitions, but no phase coexistence is observed.^27^ This contrasts with our results discussed below. The *Imma* structure is almost impossible to discern from the *Pnma* structure using laboratory X-ray diffraction data.^12^ In this manuscript, we have elected to use the *Imma* structural description for the Ln = Pr–Sm materials.

The main impact of this work is the systematic exploration of the oxygen cycling capacity of the Ln_0.5_Sr_0.5_CoO_3−δ_ perovskites using thermogravimetric analysis. The cycling capacity is largely governed by the highest accessible oxygen content (δ_min_), which decreases with decreasing Ln size. By contrast, the maximum amount of oxygen that can be removed (δ_max_) is fairly constant. In terms of specific compositions, Ln = Pr–Sm have perovskite structures with octahedral tilting and good oxygen storage capacities. Smaller Ln = Gd–Y form with reduced Ln content and have A-site ordered perovskite structures and a higher degree of oxygen nonstoichiometry, which limits their oxygen cycling performance. Neutron powder diffraction on Pr_0.5_Sr_0.5_CoO_3−δ_ reveals a transition to the basic cubic perovskite structure on heating and confirms the oxygen loss from thermogravimetric measurements.

## Experimental Methods

Polycrystalline Ln_1–*x*_Sr_*x*_CoO_3−δ_ (Ln = La, Pr, Nd, Sm, Gd, Tb, Dy, and Y) samples were prepared on a 5 g scale by the solid-state reaction method. Here, *x* = 0.5 for Ln = La–Tb and *x* = 0.67 for Ln = Dy and Y. All reported measurements and analysis were undertaken on the same samples. Dy_2_O_3_ (Alfa Aesar, 99.99%), Gd_2_O_3_ (Alfa Aesar, 99.99%), La_2_O_3_ (Sigma-Aldrich, 99.999%), Nd_2_O_3_ (Alfa Aesar, 99.99%), Pr_6_O_11_ (Alfa Aesar, 99.99%), Sm_2_O_3_ (Alfa Aesar, 99.99%), Tb_2_O_3_ (Sigma-Aldrich, 99.99%), and Y_2_O_3_ (Sigma-Aldrich, 99.999%) were used as a Ln precursor. Stoichiometric amounts of Ln oxides, Co_3_O_4_ (Alfa Aesar, 99.9985%), and SrCO_3_ (Alfa Aesar, 99.997%) were mixed using a mortar and pestle and heated in a muffle furnace for 12 h at 1000 °C. The obtained calcined powders were ground together and cold pressed. Pellets of the resulting mixture were sintered in air at 1100 °C for 12 h with heating and cooling rates of 10 °C min^–1^. The average oxidation state of Co in the prepared samples was evaluated by iodometric titration. Aqueous 4 mol L^–1^ HCl solution was saturated by bubbling argon gas through the solution for a minimum of 30 min, preventing oxidation of iodide ions by air. 1 g of KI and ∼20 mg of perovskite oxide were dissolved in the HCl solution and titrated by 0.01 mol L^–1^ aqueous Na_2_S_2_O_3_ solution under an argon atmosphere. The oxygen content of the samples was calculated from the Co oxidation state and is reported in Table 1. The reported error is based on the standard deviation of three concordant measurements.

**Table 1:** Comparison of Unit Cell Parameters for Ln_0.5_Sr_0.5_CoO_3−δ_ (Ln = La, Pr, Nd, and Sm), Ln_0.4_Sr_0.6_CoO_3−δ_ (Ln = Gd and Tb), and Ln_0.3_Sr_0.7_CoO_3−δ_ (Ln = Dy and Y) Obtained from Rietveld Refinement of XRD Data

		lattice parameters (Å)				
perovskite oxides	space group	*a*	*b*	*c*	volume (Å^3^)	supercell
La_0.5_Sr_0.5_CoO_3–__δ_	*R*3̅*c*	5.4283(3)		13.2594(3)	338.37(3)	√2 × √2 × 2√3
Pr_0.5_Sr_0.5_CoO_3−δ_	*R*3̅*c*	5.4243(8)		13.1672(8)	335.51(7)	√2 × √2 × 2√3
Pr_0.5_Sr_0.5_CoO_3−δ_	*Imma*	5.3990(4)	7.6203(5)	5.4402(3)	223.82(3)	√2 × 2 × √2
Nd_0.5_Sr_0.5_CoO_3−δ_	*Imma*	5.3723(6)	7.5906(7)	5.4239(6)	221.17(7)	√2 × 2 × √2
Sm_0.5_Sr_0.5_CoO_3−δ_	*Imma*	5.3628(8)	7.583(1)	5.4227(8)	220.53(9)	√2 × 2 × √2
Gd_0.5_Sr_0.5_CoO_3−δ_	*I*4/*mmm*	7.5719(5)		15.325(1)	878.660	2 × 2 × 4
Tb_0.5_Sr_0.5_CoO_3−δ_	*I*4/*mmm*	7.5829(6)		15.333(1)	881.6(2)	2 × 2 × 4
Dy_0.3_Sr_0.7_CoO_3−δ_	*I*4/*mmm*	7.6163(4)		15.306(1)	887.9(1)	2 × 2 × 4
Y_0.3_Sr_0.7_CoO_3−δ_	*I*4/*mmm*	7.6197(2)		15.3090(6)	888.8(1)	2 × 2 × 4

Thermogravimetric analysis (TGA) was performed on ∼200 mg of each of the prepared Ln_1–*x*_Sr_*x*_CoO_3−δ_ samples using a Linseis STA 1600 instrument. Data were collected under flowing N_2_ gas (oxygen free nitrogen, BOC, flow rate ∼100 cm^3^ min^–1^). Heating and cooling rates were set at 10 °C min^–1^ with repeated temperature cycling between 300 and 1000 °C. Samples showed reproducible oxygen loss/gain on heating/cooling, revealing the presence of oxygen in the chamber. No direct measurement of the oxygen partial pressure (*p*O_2_) was made. However, the loss/gain data directly follow temperature, with higher temperatures leading to lower *p*O_2_ and reducing conditions.

Laboratory X-ray powder diffraction (XRD) data were collected using a Bruker D8 Advance diffractometer in reflection geometry with monochromated Cu Kα_1_ radiation (Ln = La, Pr, Nd, Sm, Dy, and Y). A Stoe Stadi MP diffractometer in transmission geometry with monochromated Mo Kα_1_ radiation, avoiding X-ray fluorescence from Co, was used to determine the structures of the Ln = Gd and Tb materials. Rietveld quality data sets were collected over 7 h on the Cu source, or over 16 h on the Mo source. Rietveld analysis of XRD data was carried out using the GSAS II software.^28,29^ Tabulated ionic radii for Ln^3+^ and Sr^2+^ with 8-fold coordination were used plot trends across the Ln_1–*x*_Sr_*x*_CoO_3−δ_ materials.^30^ Crystal structures were visualized using the VESTA package.^31^

Neutron powder diffraction (NPD) data on Pr_0.5_Sr_0.5_CoO_3−δ_ were collected on the D2B diffractometer at the Institut Laue-Langevin (ILL), Grenoble, France. Variable-temperature measurements were undertaken on 10 g of material between RT and 900 °C under N_2_ gas flow (oxygen free nitrogen, BOC, flow rate 50 cm^3^ min^–1^). The incident wavelength was λ = 1.5943 Å with data recorded between 10° < 2θ < 160°. Data were collected on an empty sample holder at RT, 450, and 750 °C, and these patterns were used to fix the background in the Rietveld fits of the Pr_0.5_Sr_0.5_CoO_3−δ_ sample. Rietveld analysis was again carried out using the GSAS II program. The thermal displacement parameters of Pr and Sr were refined using isotropic values at all temperatures. All atomic site occupancies in the mixed phase *R*3*c*/*Imma* samples were fixed at unity because of correlations between the site occupancies in the two phases, which led to unstable refinements. This occurred despite both phases being present in large amounts. Bond valence sums (BVS) were used to calculate the valence of atoms within their local environment. BVS parameters *r*_0_ = 1.715 and *B* = 0.37 were used for Co.^23^ At elevated temperatures, a thermal expansion correction to the BVS parameters was applied. To this end, a linear expansion coefficient α = 1.61 × 10^–5^ K^–1^ was obtained from the lattice parameter between RT and 450 °C, where limited chemical reduction occurs (Table S1 and Figure S1). This value is in good agreement with related cobalt oxide perovskite materials.^1,23,24^ Bond valence site energy (BVSE) calculations were performed using the SoftBV program.^32^ For the BVSE calculations, the experimental lattice parameters and ideal atomic coordinates based on the simple cubic perovskite structure were used.

## 2 Results and Discussion

### 2.1 Structural Evolution

Laboratory XRD was used to confirm phase formation and elucidate structural changes of the prepared Ln_0.5_Sr_0.5_CoO_3−δ_ (Ln = La, Pr, Nd, Sm, Gd, and Tb) and Ln_0.3_Sr_0.7_CoO_3−δ_ (Ln = Dy and Y) materials. Due to the low sensitivity of X-rays to light elements, it is difficult to unambiguously determine the octahedral tilt system using XRD. Our initial Rietveld fits used published atomic coordinates, and only lattice parameters were extracted. An overview of the fitted lattice parameters is presented in Table 1 and in Figure 2a,b. The Rietveld fits are shown in Figures S2 and S3. La_0.5_Sr_0.5_CoO_3−δ_ was successfully fitted using the *R*3̅*c* structure (using a hexagonal unit cell description). This composition has the highest normalized unit cell volume and similar pseudocubic lattice parameters *a*_p_ = 3.8277(1) Å and *c*_p_ = 3.8384(2) Å (Figure 2a,b). Ln = Pr has an ∼50:50 mixture of the *R*3̅*c* and *Imma* structures with similar normalized unit cell volumes but somewhat different pseudocubic lattice parameters (Table 1 and Figure 2b). The *R*3̅*c* to *Imma* transition is of the first order from Landau theory, consistent with the observed phase coexistence.^14^ Ln = Nd and Sm have the *Imma* structure with nearly identical *a* and *b* lattice parameters for the Sm composition, suggesting an imminent transition to a tetragonal structure. Indeed, for Ln = Gd and Tb, a tetragonal unit cell was found in initial refinements.

**Figure 2:**
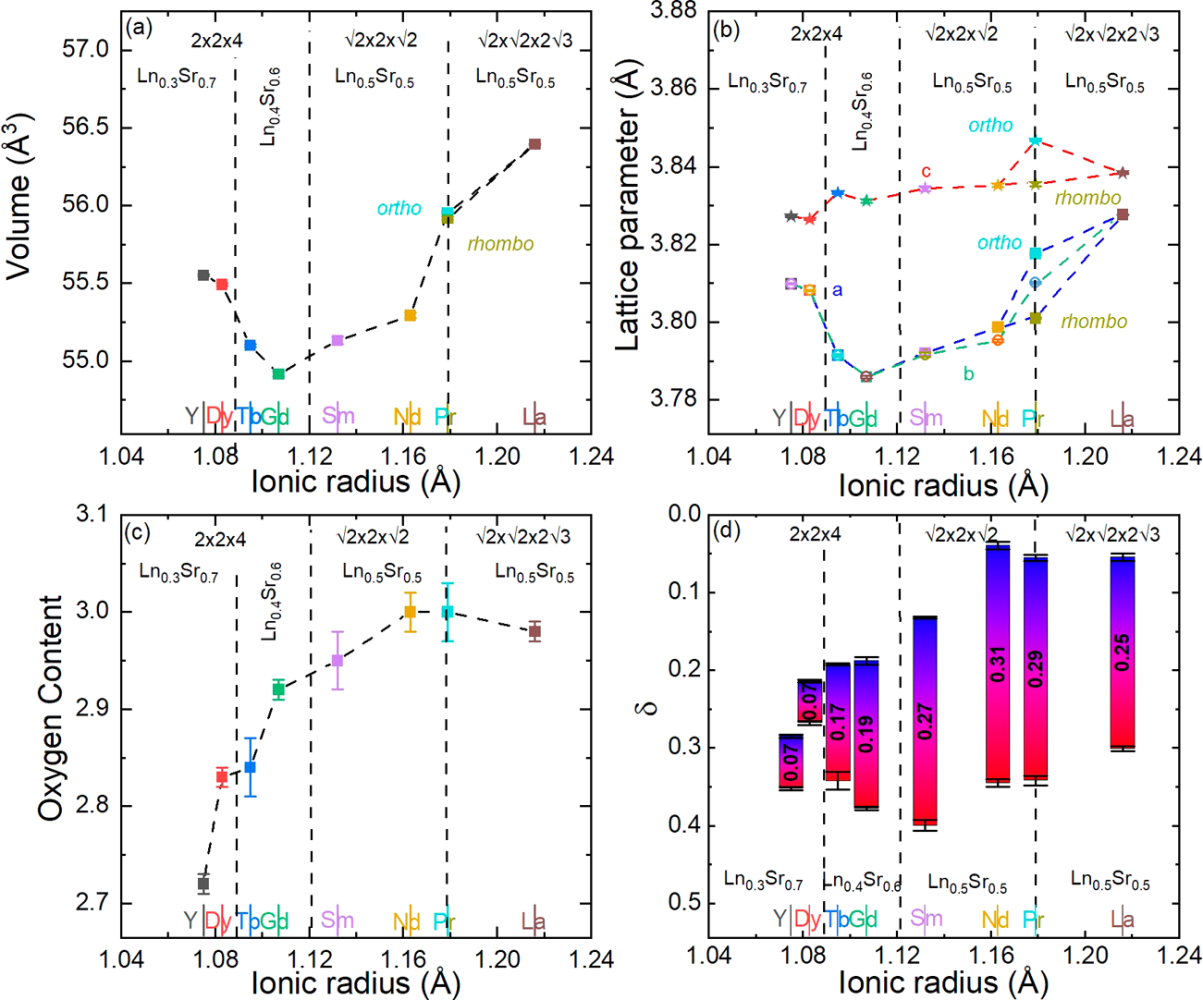
Structural, oxygen content, and cycling data for Ln_0.5_Sr_0.5_CoO_3−δ_ (Ln = La, Pr, Nd, and Sm), Ln_0.4_Sr_0.6_CoO_3−δ_ (Ln = Gd and Tb), and Ln_0.3_Sr_0.7_CoO_3−δ_ (Ln = Dy and Y) plotted against the ionic radius of lanthanides: (a) normalized unit cell volume; (b) normalized pseudocubic lattice parameters *a*, *b*, and *c*; (c) oxygen content obtained by iodometric titration; (d) changes in the oxygen content under cycling between 300 and 1000 °C.

To obtain data suitable for full structural analysis, a second set of XRD data was collected using a Mo X-ray source and analyzed using Rietveld analysis. This confirmed that the structures of these materials are described by the *I*4/*mmm* cell reported for the Sr-rich Ln_0.3_Sr_0.7_CoO_3−δ_ materials with Ln = Dy and Y. Superstructure reflections of that structure type are clearly present in the XRD data sets (Figure S3). Refinement of Ln/Sr ratios on the A-sites yields an approximate Ln_0.4_Sr_0.6_CoO_3−δ_ composition with one A-site fully occupied by Sr with the other sites 70/30 and 50/50 occupied for Ln = Gd and both sites near 50/50 mixed for Ln = Tb (see Tables S2 and S3 for full structural information). This is different from the Ln_0.3_Sr_0.7_CoO_3−δ_ materials, where Ln occupies its own site and the other two A-site positions are rich in Sr (Figure 1d). The reduced Ln content for the Gd and Tb compositions is consistent with the observation of GdCoO_3_ and Tb_2_O_3_ impurities in these samples (Figure S3). The Ln_0.3_Sr_0.7_CoO_3−δ_ (Ln = Dy and Y) compositions were confirmed to have the expected *I*4/*mmm* structure.

To summarize, the Ln_1–*x*_Sr_*x*_CoO_3−δ_ materials were successfully synthesized with structures in agreement with the literature for large Ln = La–Sm and small Ln = Dy and Y.^12,33,34^ However, intermediate-sized Ln = Gd and Tb are found to have the A-site ordered superstructure found for the *x* = 0.7 compositions but with a different A cation distribution over the A-sites and a nominal *x* = 0.6 composition.

### 2.2 Thermogravimetric Analysis

Figure 3 shows the change in oxygen content for the Ln_1–*x*_Sr_*x*_CoO_3−δ_ series over five heat–cool cycles (RT to 1000 °C, then 300–1000 °C), obtained by TGA. Values for oxygen content (δ) after synthesis were obtained by iodometric titration. These are shown in Figure 2c and are in good agreement with earlier work.^12,16,33,34^

**Figure 3:**
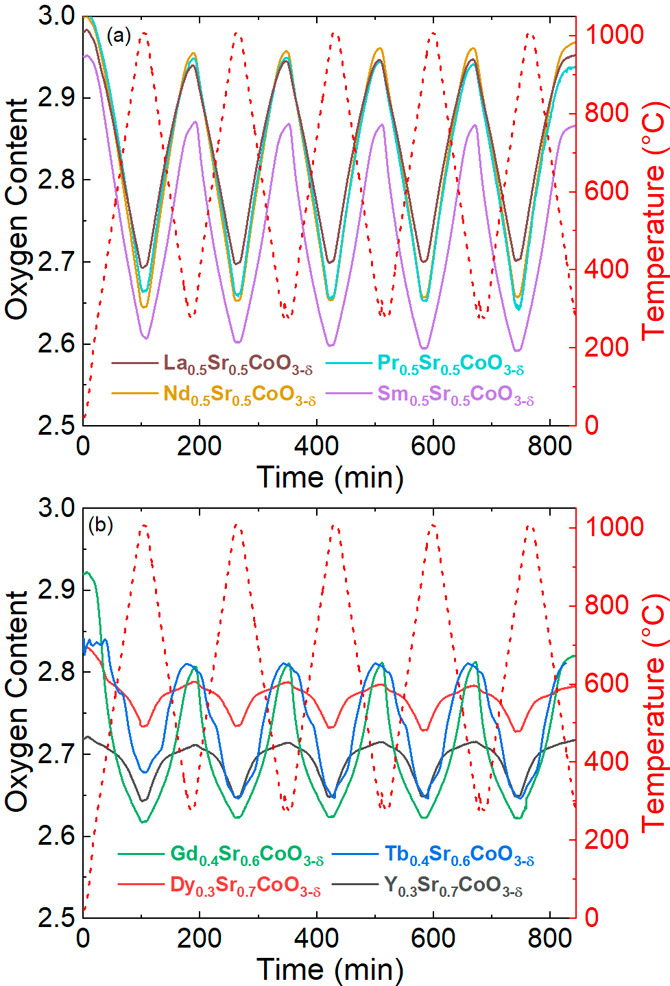
Comparison of oxygen content of (a) Ln_0.5_Sr_0.5_CoO_3−δ_ (Ln = La, Pr, Nd, and Sm), (b) Ln_0.4_Sr_0.6_CoO_3−δ_ (Ln = Gd and Tb), and Ln_0.3_Sr_0.7_CoO_3−δ_ (Ln = Dy and Y) as a function of time over five heat–cool cycles between 300 and 1000 °C, obtained by TGA.

The absolute changes in oxygen content (ΔO = δ_300C_ – δ_1000C_) from TGA during temperature cycling are shown in Figure 2d. The titration and TGA oxygen content changes are also listed in Table S4. Postcycling XRD revealed only minor changes in cell parameters and showed no degradation products (Table S5). All samples produce oxygen vacancies during heating and gain oxygen during cooling with no substantial loss of capacity over five heat–cool cycles. The TGA traces for Ln = La–Sm, with low initial δ_min_ ≤ 0.05 after synthesis, show near linear loss and gain of oxygen, i.e., following the temperature profile during heating and cooling (Figure 3a). This reveals that the *p*O_2_ in the TGA chamber is determined by temperature and that oxygen is present in sufficient quantity to oxidize the sample on cooling, with the lower *p*O_2_ on heating causing reduction. The Ln = Gd and Tb, *x* = 0.6 compositions with δ_min_ > 0.05 after synthesis have reduced capacities for oxygen cycling, limited to values ΔO < 0.2, possibly linked to the observation of the *I*4/*mmm* superstructure (Figure 3b). The TGA profile for these samples does not directly follow the temperature at low temperatures, reflecting the inability of these samples to fully oxidize. Careful inspection reveals different rates of mass change, with higher temperatures leading to faster loss/gain. This likely reflects the inability of these samples to fully oxidize, consistent with the higher level of oxygen vacancies after synthesis. The *x* = 0.7 Ln = Dy and Y compositions with the same *I*4/*mmm* superstructure have the highest δ_min_ after synthesis and the lowest Δ*O*. For example, δ_min_ = 0.28(1) and Δ*O* = 0.07 (0.6 wt %) for Y_0.3_Sr_0.7_CoO_3−δ_. The TGA profile for these samples does not follow temperature well, illustrating limited oxygen cycling in a narrower range of temperature (*p*O_2_).

The largest oxygen storage capacities Δ*O* = 0.25–0.3 are therefore found for the larger Ln = La – Sm with tilted perovskite structures without A-site ordering. Ln = Nd has the highest capacity Δ*O* = 0.3, corresponding to 2.2 wt %. The main difference between large and small Ln samples is in the oxygen content after synthesis (δ_min_), which is lower for the smaller Ln and forms a an upper bound for maximum achievable oxygen content. Large and small Ln achieve similar δ_max_ ∼0.35 showing that the main variation between samples is linked to δ_min_. A similar trend was observed for the layered LnBaCo_2_O_6−δ_ perovskite materials.^24^ Large size differences between Ln/Ba result in reduced oxygen content after synthesis and reduced oxygen cycling capacity.

### 2.3 NPD of Pr_0.5_Sr_0.5_CoO_3−δ_

As observed in XRD, the Ln = Pr sample (prepared on a 10 g scale) was found to consist of a two-phase mixture of 40% *Imma* and 53% *R*3̅*c* phases, with the remainder being small made up of small amounts of Co (5 wt %) and Pr_2_O_3_ (2 wt %) (Table S6).

The presence of metallic Co indicates that some reduction has taken place at high temperature during preparation of the sample. No evidence for cation nonstoichiometry was observed for the perovskite phases from Rietveld analysis. The presence of two phases agrees with the initial sample prepared for the TGA analysis but contrasts with the NPD study in the literature, where no phase coexistence was observed.^27^ Those samples were prepared in oxygen flow with slow cooling (60 °C/h), suggesting that the phase coexistence in our samples occurs due to slow equilibration during cooling. Refined structural parameters are given in Table 2, and the quality of the fit is shown in Figure 4a. The *Imma* phase has Co–O–Co angles near 170° consistent with the *a* ^–^ *b* ^0^ *a* ^–^ tilt system, with similar tilts around the pseudocubic *a* and *c* axes (Table 2).^14,15^ The CoO_6_ octahedra are slightly compressed in the *Imma* structure (*d*_apical_/*d*_basal_ = 1.910 Å/1.920 Å; Table 2). The thermal atomic displacement parameters (ADPs) for Co and O1 exhibit higher values within the CoO_2_ plane (Figure S4 and Table S7), whereas the O2 position shows the strongest displacement along the apical axis. The *R*3̅*c* phase has symmetric CoO_6_ octahedra with bond distances of 1.920(1) Å, comparable to the apical bond distance in the *Imma* phase (Table 2 and Figure 4f). The largest O ADPs were observed along the *a* and *c* directions. In all cases, the ADPs have an ellipsoidal disk shape characteristic of the curved path for oxygen migration in perovskite materials.^26^

**Figure 4:**
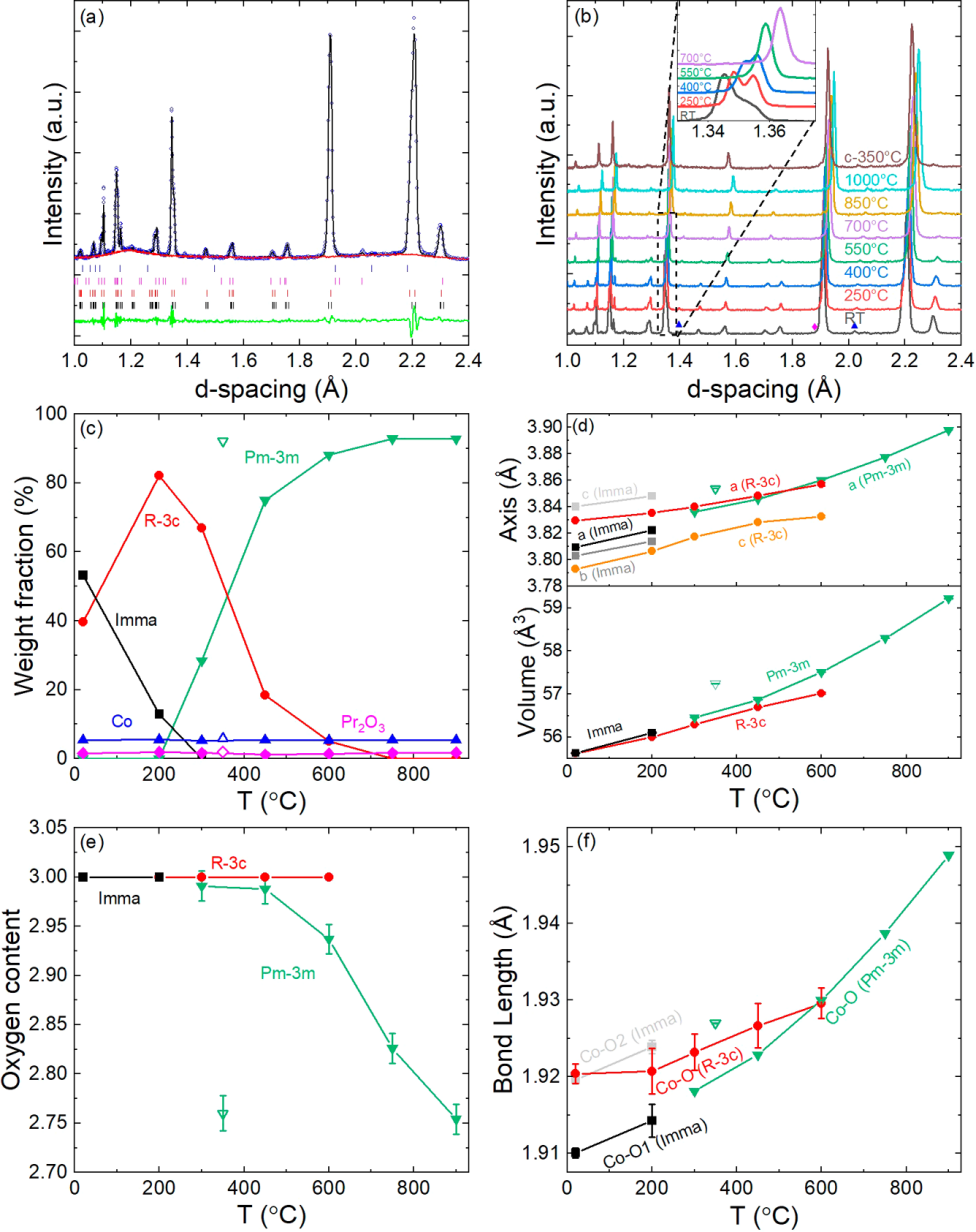
(a) Rietveld fit to room-temperature NPD data of Pr_0.5_Sr_0.5_CoO_3−δ_. Bragg peak positions of orthorhombic (*Imma*) and rhombohedral (*R*3̅*c*) Pr_0.5_Sr_0.5_CoO_3−δ_; the impurities Co and Pr_2_O_3_ are indicated with black, red, blue, and magenta markers. (b) Stacked NPD patterns of Pr_0.5_Sr_0.5_CoO_3−δ_ collected between RT and 900 °C on heating and after cooling under N_2_ flow. Impurity peaks of Co and Pr_2_O_3_ are identified by triangles and diamonds, respectively. Resultant changes in (c) the weight fractions of the Pr_0.5_Sr_0.5_CoO_3−δ_ phases and impurities, (d) normalized lattice parameters and unit cell volumes, (e) oxygen content, and (f) Co–O bond lengths of different Pr_0.5_Sr_0.5_CoO_3−δ_ structures. Data on cooling are indicated by open symbols.

**Table 2:** Rietveld Fit Parameters for Pr_0.5_Sr_0.5_CoO_3−δ_ between RT and 900 °C upon Heating and at 350 °C after Cooling^a^

	RT		200 °C		300 °C		450 °C		600 °C		750 °C	900 °C	350 °C-c
space group	*Imma*	*R*3̅*c*	*Imma*	*R*3̅*c*	*R*3̅*c*	*Pm*3̅*m*	*R*3̅*c*	*Pm*3̅*m*	*R*3̅*c*	*Pm*3̅*m*	*Pm*3̅*m*	*Pm*3̅*m*	*Pm3̅m*
*a* (Å)	5.3874(2)	5.4160(3)	5.4058(4)	5.4241(2)	5.4307(2)	3.8362(1)	5.4423(5)	3.8457(6)	5.455(2)	3.8600(6)	3.8775(7)	3.8978(1)	3.8539(1)
*b* (Å)	7.6060(2)		7.6280(6)										
*c* (Å)	5.4308(2)	13.1392(4)	5.4424(7)	13.1859(3)	13.2236(3)		13.2612(7)		13.277(3)				
*V* (Å^3^)	222.535(1)	333.78(3)	224.417(5)	335.97(2)	337.74(2)	56.457(6)	340.16(4)	56.874(3)	342.1(1)	57.512(3)	58.296(3)	59.220(5)	57.243(5)
Pr/Sr (*z*)	0.004(1)		0.002(4)										
O1 (*x*)		0.461(2)		0.468(5)	0.470(7)		0.472(5)		0.474(4)				
O1 (*z*)	0.467(1)		0.469(4)										
O2 (*y*)	0.0218(3)		0.020(1)										
*d*_Co–O1_ (Å)	1.9101(6)	1.920(1)	1.914(2)	1.921(3)	1.923(2)	1.9181(7)	1.927(3)	1.9228(3)	1.930(2)	1.9300(3)	1.9387(4)	1.9489(5)	1.9270(6)
*d*_Co–O2_ (Å)	1.9196(2)		1.9239(9)										
Co–O1–Co (deg)	169.14(2)	167.38(6)	170.00(3)	169.66(7)	170.25(7)		171.07(8)		171.7(1)				
Co–O2–Co (deg)	170.00(3)		170.81(4)										
Occ O1	1.0	1.0	1.0	1.0	1.0	0.997(5)	1.0	0.996(5)	1.0	0.979(5)	0.942(5)	0.918(5)	0.920(6)
Occ O2	1.0		1.0										
O cont.	3.00	3.00	3.00	3.00	3.00	2.99(2)	2.99	2.98(2)	2.99	2.94(2)	2.83(2)	2.75(2)	2.76(2)
Co^*x*+^	3.5	3.5	3.5	3.5	3.5	3.48(3)	3.5	3.48(3)	3.5	3.37(3)	3.15(3)	3.01(3)	3.02(4)
weight fraction (%)	53.3	39.7	13.0	82.1	67.0	28.4	18.4	75.0	5.1	88.2	92.9	92.8	92.1
*wR*_P_ (%)	3.8		4.9		4.5		4.1		4.0		3.9	3.9	5.2
*R*_F_ (%)	3.4	2.9	5.7	5.0	5.5	3.7	8.5	4.0	45	4.6	4.9	7.5	4.2

a Parameters were refined by using three models: √2*a*_p_ × 2*a*_p_ × √2*a*_p_ orthorhombic (*Imma*) supercell with sites Pr 4e (0 0.25 *z*), Sr 4e (0 0.25 *z*), Co 4b (0 0 0.5), O1 4e (0 0.25 *z*), O2 8g (0.25 *y* 0.75); √2*a*_p_ × √2*a*_p_ × 2√3*a*_p_ rhombohedral (*R*3̅*c*) supercell with sites Pr 6a (0 0 0.25), Sr 6a (0 0 0.25), Co 6b (0 0 0), O 18e (*x* 0 0.25); and *a*_p_ × *a*_p_ × *a*_p_ cubic (*Pm*3̅*m*) cell with sites Pr 1b (0.5 0.5 0.5), Sr 1b (0.5 0.5 0.5), Co 1a (0 0 0), O 3d (0 0 0.5).

Stacked NPD patterns collected between RT and 900 °C during heating and cooling under flowing N_2_ are shown in Figure 4b. These show not only thermal expansion of the lattice parameters but also phase coexistence over a large temperature domain (Figure 4c). Two structural phase changes: from *Imma* → *R*3̅*c* between RT and 300 °C and from *R*3̅*c* → *Pm*3̅ m between 200 and 750 °C, are observed. Phase coexistence is expected for the first-order nature of the change in tilt systems (*a* ^–^ *b* ^0^ *a* ^–^ → *a* ^–^ *a* ^–^ *a* ^–^ → *a* ^0^ *a* ^0^ *a* ^0^) but contrasts with the literature.^27^ No accordance between any lattice parameter can be identified in the first phase change between RT and 300 °C (Figure 4d). For the second phase change, between 200 and 750 °C, the normalized *a* lattice parameter is similar, with the *c* axis showing an increase. For both domains of phase coexistence, the cell volumes are more similar than the discontinuity in lattice parameters, which is in keeping with the large temperature domain over which this occurs, with both polymorphs having comparable lattice energies (Figure 4d).

The fitted overall oxygen content changes from no vacancies (δ = 0.01(2)) at 300 °C to δ = 0.25(2) at 1000 °C (Figure 4e), in good agreement with the TGA data (ΔO = 0.29(2)). Substantial oxygen loss occurs above 450 °C at which point the cubic phase is the dominant component of the sample. This is comparable to literature data on the Ln = La materials that show little oxygen loss until around 400 °C.^7,35^ During cooling to 350 °C under flowing N_2_, the sample does not regain oxygen and has a significantly increased lattice parameter (Figure 4d). The lack of oxygen uptake contrasts with the TGA experiment and signals a lower *p*O_2_ on cooling, which we attribute to a tighter sample chamber as the same oxygen free nitrogen gas was used. At this low oxygen content (δ = 0.24(2)), the sample maintains the cubic perovskite structure down to 350 °C with no evidence for a phase transition to the R 3̅ c structure.

Bond valence sum (BVS) calculations were undertaken to monitor the oxidation state of Co as the sample is heated under flowing N_2_. This uses the calculated bond distances,^36^ with the rapid increase in the Co–O bond distance (Figure 4f) indicating a substantial reduction of Co. At RT, the BVS of Co is ∼ 3.5, in both *Imma* and R 3̅ c structures (Table S8), in good agreement with the expected oxidation state (+3.5 for δ = 0). As outlined in the experimental methods sections, a correction for regular thermal expansion was applied to the BVS parameters,^37^ enabling the impact of chemical reduction to be separated from regular thermal (vibration-based) expansion. At higher temperatures, the formation of oxygen vacancies leads to a decrease in the Co oxidation state, reaching ∼3.1 at 1000 °C, which is in good agreement with the TGA (δ = 0.4, Co^3.2+^) and the +3 oxidation state from the fitted NPD composition. It is also comparable to literature data on the *x* = 0.6, *A* = Sr system, where the Co bond valence decrease from 3.3+ to 2.9+ at 1000 °C.^26^ Furthermore, the BVS values of for the O anions and A-site cations are close to the expected oxidation states of −2 and +2.5, respectively. Overall, the BVS confirm the reduction of the Co cations as the sample is heated and oxygen vacancies are created.

## 3 Discussion

This work presents a systematic investigation of oxygen cycling in near half-doped Ln_1–*x*_Sr_*x*_CoO_3−δ_ perovskites. The structures for large Ln = La–Sm with *x* = 0.5 and small Ln = Dy and Y with *x* = 0.7 agree with those reported in the literature. Intermediate Ln = Gd and Tb samples were found to have a higher Sr content (*x* = 0.6) and the same A-site ordered superstructure found for Ln = Dy and Y. However, the ordering pattern of the Ln/Sr cations is different between the *x* = 0.6 and *x* = 0.7 samples, with either Sr or Ln fully occupying one of the A-sites. Two factors are at play in the structural transition toward A-site ordering:^16^ the ordering itself is driven by the increasing size difference between Ln and Sr; in parallel decreasing the average A-site radius reduces the structural stability,^38^ which in turn leads to the increasing Sr content from *x* = 0.5 to *x* = 0.6 for Ln = Gd and Tb and *x* = 0.67 for Ln = Dy and Y.

The oxygen cycling capacity is found to be highest for Ln = La–Sm with ΔO = 0.2–0.3 mol and ∼ 2 wt % between 300 and 1000 °C. The smaller Ln have a lower stable oxygen content (higher δ_min_) after materials synthesis and cycle in a narrow range around a lower overall oxygen content (Figure 2d). For the larger Ln, the TGA trace mirrors the temperature profile, whereas at least two different domains are apparent for the smaller Ln (Figure 3), which reflects the inability of these materials to reach higher oxygen content (low δ_min_). Overall, these pseudocubic perovskites show a comparable reduction in oxygen cycling capacity to the layered LnBaCo_2_O_6−δ_ materials.^24^ This reveals that both material systems have a similar change in structural stability as the Ln size is reduced. Oxygen vacancy formation involves removal of a neutral oxygen and reduction of the cobalt metal, with the intrinsic stability of the structure governing the vacancy concentration.^39^ The similar TGA mass changes for the layered and pseudocubic perovskites with decreasing Ln size suggests that the intrinsic stability of the materials is similar in both systems. In the regular Ln_0.5_Sr_0.5_CoO_3−δ_ perovskites, smaller Ln destabilize the structure due to increased octahedral tilting. In the layered LnBaCo_2_O_6−δ_ system, tilting does not occur, but instead the increasing size mismatch strains the structure and reduces its stability.^3^

Neutron powder diffraction for Pr_0.5_Sr_0.5_CoO_3−δ_ reveals the presence of significant phase coexistence between between *Imma* and R 3̅ c phases and subsequently between the R 3̅ c and cubic perovskite phase. This phase coexistence largely occurs below the onset of oxygen loss near 400 °C, at which most of the sample is in the cubic perovskite form. The oxygen loss from Rietveld analysis agrees with the reduction observed in TGA, with gradual reduction of Co from +3.5 at 25 °C to +3 at 1000 °C.

In our previous work on the LnBaCo_2_O_6−δ_ system we used Bond Valence Site Energy (BVSE) calculations to map out changes to the oxygen migration pathways.^23,24^ Trial calculations on the Ln_0.5_Sr_0.5_CoO_3−δ_ samples using the fitted NPD structure for Ln = Pr and structures with ideal coordinates but lattice parameters based on the laboratory XRD data, revealed the same curved 3D migration pathways as previously observed.^26,40^ These calculations are purely elastic and allow a comparison of changes in steric effects (cation ordering, changes in cell metrics) on the migration pathways but do not consider structural distortions during migration.

Figure 5 shows the evolution of the energy barrier for migration through the bottleneck in the perovskite structure. The size of the bottleneck can be estimated from the pseudocubic lattice parameter,^26^ using (√2/4)­(*a*_p_)^2^, and is nearly constant at 5.1–5.2 Å^2^ across all compositions, including the *x* > 0.5 samples. The calculated barrier energies decrease from 2.1 eV (Ln = La) to 1.5 eV (Ln = Gd and Tb) and then increase to 2.0–2.3 eV for the *x* = 0.7 samples. These values are larger than experimentally observed (e.g., 1.4 eV for *x* = 0.5, Ln = La),^41,42^ indicating that framework flexibility is important for lowering the migration barriers. The decreasing barrier energies indicate that smaller Ln reduces steric hindrance for migration through the bottleneck that itself does not change much in size. The *x* = 0.7 compositions have increased migration barriers as these now contain 70% Sr^2+^ cations, which reduces the free space in the bottleneck area. Interestingly, the migration barriers for the *x* = 0.6 samples are the lowest calculated, despite the higher Sr content. This is tentatively explained by the ordering of the Ln/Sr cations in this structure with one of the A sites fully occupied by Sr. This separation of the largest A cation enables migrating oxide anions to avoid the most constricted pathways. Higher-quality structure data are needed to explore this further, but this observation suggests a possible route for materials development exploiting cation ordering.

**Figure 5:**
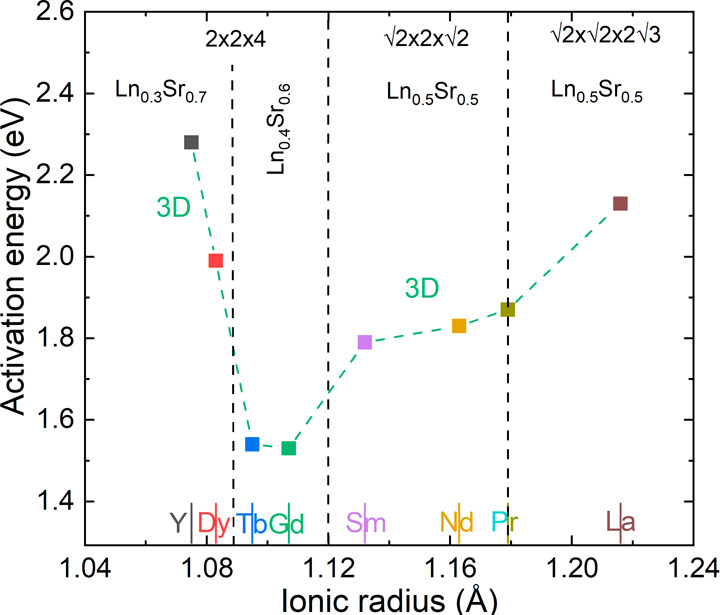
Overview of BVSE energy barriers for 3D oxygen migration for the Ln_0.5_Sr_0.5_CoO_3−δ_ (Ln = La, Pr, Nd, and Sm), Ln_0.4_Sr_0.6_CoO_3−δ_ (Ln = Gd and Tb), and Ln_0.3_Sr_0.7_CoO_3−δ_ (Ln = Dy and Y) perovskites plotted against the lanthanide ionic radius.

In summary, this study elucidates how variation of the lanthanide cation influences the oxygen cycling capacity in near half-doped Ln_0.5_Sr_0.5_CoO_3−δ_ perovskites. Increased size mismatch between Ln and Sr is shown to promote lower oxygen contents following synthesis and diminish oxygen cycling performance. Optimal capacities are therefore achieved with larger lanthanide cations (Ln = La–Sm). The pronounced reduction in capacity observed for smaller Ln is associated with the onset of A-site cation ordering and an increase in Sr content within the perovskite lattice. Neutron powder diffraction analysis of Pr_0.5_Sr_0.5_CoO_3−δ_ reveals extensive structural phase coexistence, with oxygen loss above 400 °C and selective reduction of cobalt. These findings provide a structure–property framework for understanding and tuning oxygen redox behavior in Ln_0.5_Sr_0.5_CoO_3−δ_ systems.

## Supplementary Material



## Data Availability

The research data supporting this publication can be accessed at 10.17630/b019f5c3-185d-4784-ad4d-e25a6b26ad02.

## Notes

Crystallographic information from X-ray and neutron powder diffraction analysis, results from iodometric titration, Rietveld fits to laboratory X-ray powder diffraction data, chemical and thermal lattice thermal expansion, and anisotropic atomic displacement parameters (PDF)
