# Supplementary material for: Oxygen Cycling in Half-Doped Ln1–x Sr x CoO3−δ Cobalt Perovskite Oxides and an In Situ Neutron Diffraction Study of Pr0.5Sr0.5CoO3−δ
Source: ACS Omega. 2026 May 25;11(22):32996–3004. doi: 10.1021/acsomega.6c02673 (PMC13261575; doi:10.1021/acsomega.6c02673)
Supplement: Supplementary file 1 [file ao6c02673_si_001.pdf]

Supplementary Information

for

Oxygen cycling in half-doped $Ln_{1-x}Sr_xCoO_{3-\delta}$ cobalt perovskite oxides and in-situ neutron diffraction study of $Pr_{0.5}Sr_{0.5}CoO_{3-\delta}$

Fabian Hesse,[a] Blair F. Kennedy,[b] Emmanuelle Suard,[c] Jan-Willem G. Bos*[b]

[a] *Institute of Chemical Sciences, School of Engineering and Physical Sciences, Heriot-Watt University, Edinburgh, EH14 4AS, UK.*

[b] *EaStCHEM School of Chemistry, University of St Andrews, North Haugh, St Andrews, KY16 9ST, UK.*

[c] *Institut Laue-Langevin, 71 Avenue des Martyrs, CS20156, 38042 Grenoble Cédex 9, France.*

*Email: j.w.g.bos@st-andrews.ac.uk

Table S1. Linear fitting parameters for decoupling of regular thermal expansion from reduction for $Pr_{0.5}Sr_{0.5}CoO_{3-\delta}$ between RT and 450 °C displayed in Figure S1 and thermal expansion parameter α.

parameter	
a_0 (K)	3.82
b x 10^{-5}	6.14
α (K^{-1} x 10^{-5})	1.61

Table S2. Atomic parameters for $Gd_{0.5}Sr_{0.5}CoO_{3-\delta}$ obtained from Rietveld analysis of Mo source XRD data. Lattice parameters are given in Table 1. The fitted composition is $Gd_{0.40(2)}Sr_{0.60(2)}CoO_{2.92}$ with the overall oxygen content fixed to the value from iodometric titration. Site labelling corresponds to that used in Fig. 1d in the manuscript. The sample contains a 4.5 wt% $GdCoO_3$ secondary phase.

Atom	Wykoff	x	y	z	Occ	U_{iso}
Co1	8h	0.25	0.25	0	1	0.0104(6)
Co2	8f	0.25	0.25	0.25	1	0.0104(6)
(Gd/Sr)	4e	0	0	0.1226(8)	0.67(2)/	0.0104(6)
					0.33(2)	0.0104(6)
(Gd/Sr)2	8g	0	0.5	0.1244(6)	0.47(3)/	0.0104(6)
					0.53(3)	0.0104(6)
Sr1	4e	0	0	0.6075(9)	1	0.0104(6)
O1	16m	0.23	0.23	0.121	1	0.0104(6)
O2	8i	0.197	0	0	0.84	0.0104(6)
O3	8j	0.23	0.5	0	1	0.0104(6)
O4	16n	0	0.252	0.258	1	0.0104(6)

Goodness of fit: wR_p = 8.1% R_F = 8.6%

Table S3. Atomic parameters for $Tb_{0.5}Sr_{0.5}CoO_{3-\delta}$ obtained from Rietveld analysis of Mo source XRD data. Lattice parameters are given in Table 1. The fitted composition is $Tb_{0.40(2)}Sr_{0.60(2)}CoO_{2.84}$ with the overall oxygen content fixed to the value from iodometric titration. Site labelling corresponds to that used in Fig. 1d in the manuscript. The sample contains an 8 wt% Tb_2O_3 secondary phase.

Atom	Wykoff	x	y	z	Occ	U_{iso}
Co1	8h	0.25	0.25	0	1	0.0077(7)
Co2	8f	0.25	0.25	0.25	1	0.0077(7)
(Tb/Sr)	4e	0	0	0.126(2)	0.48(3)/	0.0077(7)
					0.52(3)	0.0077(7)
(Tb/Sr)2	8g	0	0.5	0.125(2)	0.56((2)/	0.0077(7)
					0.44(2)	0.0077(7)
Sr1	4e	0	0	0.608(1)	1	0.0077(7)
O1	16m	0.23	0.23	0.121	1	0.0077(7)
O2	8i	0.197	0	0	0.68	0.0077(7)
O3	8j	0.23	0.5	0	1	0.0077(7)
O4	16n	0	0.252	0.258	1	0.0077(7)

Goodness of fit: wR_p = 5.7% R_F = 7.2%

Table S4. Oxygen content and ionic radii of $Ln_{1-x}Sr_xCoO_{3-\delta}$. Oxygen content after synthesis are from iodometric titration and changes in oxygen content are obtained by TGA between RT and 1000°C under N_2

Perovskite oxides	Oxygen content	Δ Oxygen content	Ionic radius (Å)[32]
$La_{0.5}Sr_{0.5}CoO_{3-\delta}$	2.98(1)	0.25(3)	1.216
$Pr_{0.5}Sr_{0.5}CoO_{3-\delta}$	3.00(3)	0.29(2)	1.179
$Nd_{0.5}Sr_{0.5}CoO_{3-\delta}$	3.00(2)	0.31(3)	1.163
$Sm_{0.5}Sr_{0.5}CoO_{3-\delta}$	2.95(4)	0.27(1)	1.132
$Gd_{0.5}Sr_{0.5}CoO_{3-\delta}$	2.92(1)	0.19(3)	1.107
$Tb_{0.5}Sr_{0.5}CoO_{3-\delta}$	2.84(3)	0.17(5)	1.095
$Dy_{0.3}Sr_{0.7}CoO_{3-\delta}$	2.83(1)	0.07(3)	1.083
$Y_{0.3}Sr_{0.7}CoO_{3-\delta}$	2.72(1)	0.07(2)	1.075

Table S5. Lattice parameters and cell volumes of $Ln_{1-x}Sr_xCoO_{3-\delta}$ (Ln = La, Pr, Nd, Sm, Gd, Tb, Dy, Y) after five heat-cool cycles (RT-1000 °C) under nitrogen determined by XRD.

Perovskite oxides	Space group	Lattice parameters (Å)			Volume (Å^3)	Volume change (%)
		a	b	c		
$La_{0.5}Sr_{0.5}CoO_{3-\delta}$	R-3c	5.4246(2)		13.2553(3)	337.79(2)	0.17
$Pr_{0.5}Sr_{0.5}CoO_{3-\delta}$	R-3c	5.4181(6)		13.1639(6)	334.67(5)	0.25
$Pr_{0.5}Sr_{0.5}CoO_{3-\delta}$	Imma	5.3995(4)	7.6150(4)	5.4310(4)	223.3(3)	0.23
$Nd_{0.5}Sr_{0.5}CoO_{3-\delta}$	Imma	5.376(1)	7.598(2)	5.421(2)	221.4(2)	-0.12
$Sm_{0.5}Sr_{0.5}CoO_{3-\delta}$	Imma	5.374(1)	7.589(2)	5.396(2)	220.1(2)	-0.16
$Gd_{0.5}Sr_{0.5}CoO_{3-\delta}$	I4/mmm	7.5718(5)		15.324(1)	878.66(3)	-0.32
$Tb_{0.5}Sr_{0.5}CoO_{3-\delta}$	I4/mmm	7.5821(6)		15.336(1)	881.6(2)	-0.08
$Dy_{0.3}Sr_{0.7}CoO_{3-\delta}$	I4/mmm	7.6179(4)		15.3057(9)	888.2(1)	-0.04
$Y_{0.3}Sr_{0.7}CoO_{3-\delta}$	I4/mmm	7.6192(4)		15.3066(5)	888.59(9)	0.03

Table S6. Impurity weight fractions and $Pr_{0.5}Sr_{0.5}CoO_{3-\delta}$ main phases measured at different temperatures (RT - 900 °C) upon heating and after cooling under N_2 flow.

Impurity (wt.%)	RT	200°C	300°C	450°C	600°C	750°C	900°C	350°C-c
Imma	53.3(2)	13.0(3)	0	0	0	0	0	0
$R\bar{3}c$	40.0(4)	81.9(3)	67.3(3)	18.3(4)	4.5(6)	0	0	0
$Pm\bar{3}m$	0	0	28.4(5)	75.0(6)	88.2(8)	92.9(9)	93(1)	92.1(9)
Co	5.5(2)	5.4(2)	5.3(2)	5.5(2)	5.4(2)	5.4(2)	5.4(2)	5.9(2)
Pr_2O_3	1.6(3)	1.9(3)	1.7(3)	1.1(3)	1.4(3)	1.7(3)	1.8(3)	1.9(3)

Table S7. ADP (Å² x 100) for orthorhombic, rhombohedral and cubic $Pr_{0.5}Sr_{0.5}CoO_{3-\delta}$ between RT and 900 °C upon heating and at 350 °C after cooling.

ADP (Å² x 100)	RT		200°C		300°C		450°C		600°C		850°C	900°C	350°C-c
space group	Imma	$R\bar{3}c$	Imma	$R\bar{3}c$	$R\bar{3}c$	$Pm\bar{3}m$	$R\bar{3}c$	$Pm\bar{3}m$	$R\bar{3}c$	$Pm\bar{3}m$	$Pm\bar{3}m$	$Pm\bar{3}m$	$Pm\bar{3}m$
U(Pr/Sr)	0.5(1)	0.4(1)	0.7(3)	0.5(1)	0.6(1)	1.8(2)	0.6(2)	2.0(1)	0.7(1)	2.2(1)	2.9(1)	3.4(1)	1.1(1)
U_{11}(Co)	1.4(5)	1.0(1)	2(1)	1.2(1)	1.3(2)	1.0(4)	1.8(4)	1.1(1)	2.9(3)	1.1(1)	1.6(1)	2.1(1)	2.1(1)
U_{22}(Co)	0.8(2)		1.3(8)										
U_{33}(Co)	2.2(3)	0.6(2)	3(1)	0.6(2)	1.0(2)		1.5(6)		1.8(9)				
U_{12}(Co)		0.5(1)		0.6(1)	0.7(1)		1.0(2)		1.5(5)				
U_{23}(Co)	0.5(3)		0.6(4)										
U_{11}(O1)	3.4(4)	0.8(3)	6(1)	1.2(3)	1.4(4)	3.2(2)	1.8(7)	3.9(1)	2(1)	5.1(1)	5.9(1)	7.2(1)	5.5(1)
U_{22}(O1)	0.6(2)	0.4(2)	1.5(5)	1.7(2)	2.5(2)		3.4(7)		5(2)				
U_{33}(O1)	4.2(4)	1.0(1)	8(1)	1.8(1)	2.1(1)	1.1(2)	2.5(2)	1.4(1)	2.8(8)	1.6(1)	1.9(1)	2.5(1)	1.5(1)
U_{12}(O1)		0.2(1)		0.8(1)	1.3(1)		1.7(3)		2.3(9)				
U_{13}(O1)		0.1(1)		0.2(1)	0.2(1)		0.2(1)		0.3(1)				
U_{23}(O1)		0.3(2)		0.5(2)	0.5(3)		0.5(3)		0.6(4)				
U_{11}(O2)	2.1(2)		3.8(4)										
U_{22}(O2)	1.3(1)		2.4(5)										
U_{33}(O2)	1.0(1)		1.2(8)										
U_{13}(O2)	0.6(1)		0.8(6)										

Table S8. BVSs for $Pr_{0.5}Sr_{0.5}CoO_{3-\delta}$ between RT and 900 °C upon heating and at 350 °C after cooling. O, R and C are presenting the crystal structures of orthorhombic (Imma), rhombohedral (R $\bar{3}$c) and cubic (Pm$\bar{3}$m) phases, respectively.

BVS	RT		200°C		300°C		450°C		600°C		850°C	900°C	350°C-cool
	O	R	O	R	R	C	R	C	R	C	C	C	C
Pr	2.74	2.79	2.71	2.74	2.72	2.61	2.83	2.60	2.96	2.52	2.38	2.27	2.34
Sr	2.70	2.73	2.68	2.70	2.69	2.60	2.76	2.59	2.84	2.51	2.38	2.27	2.34
Co	3.51	3.47	3.51	3.51	3.51	3.55	3.43	3.54	3.35	3.45	3.28	3.14	3.21
O1	2.10	2.08	2.09	2.08	2.07	2.06	2.07	2.05	2.08	2.03	2.00	1.97	2.01
O2	2.06		2.06										

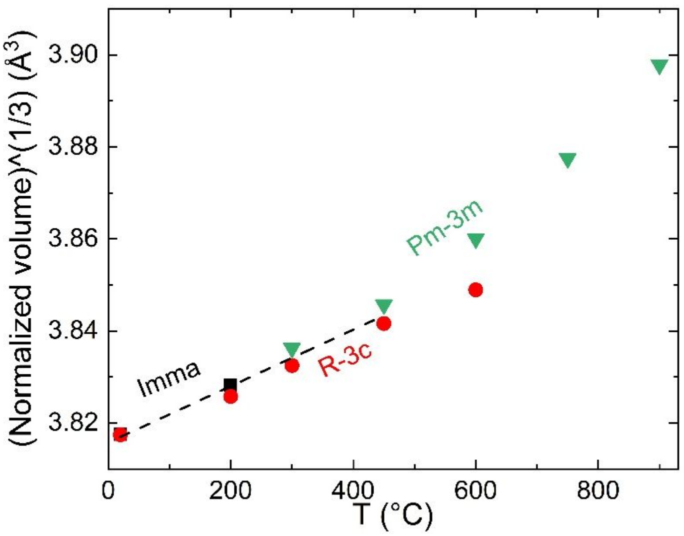

Figure S1. Linear volume fits of $Pr_{0.5}Sr_{0.5}CoO_{3-\delta}$ between RT and 450 °C to decouple regular thermal expansion from reduction and to determine the correction term α (see Table S1).

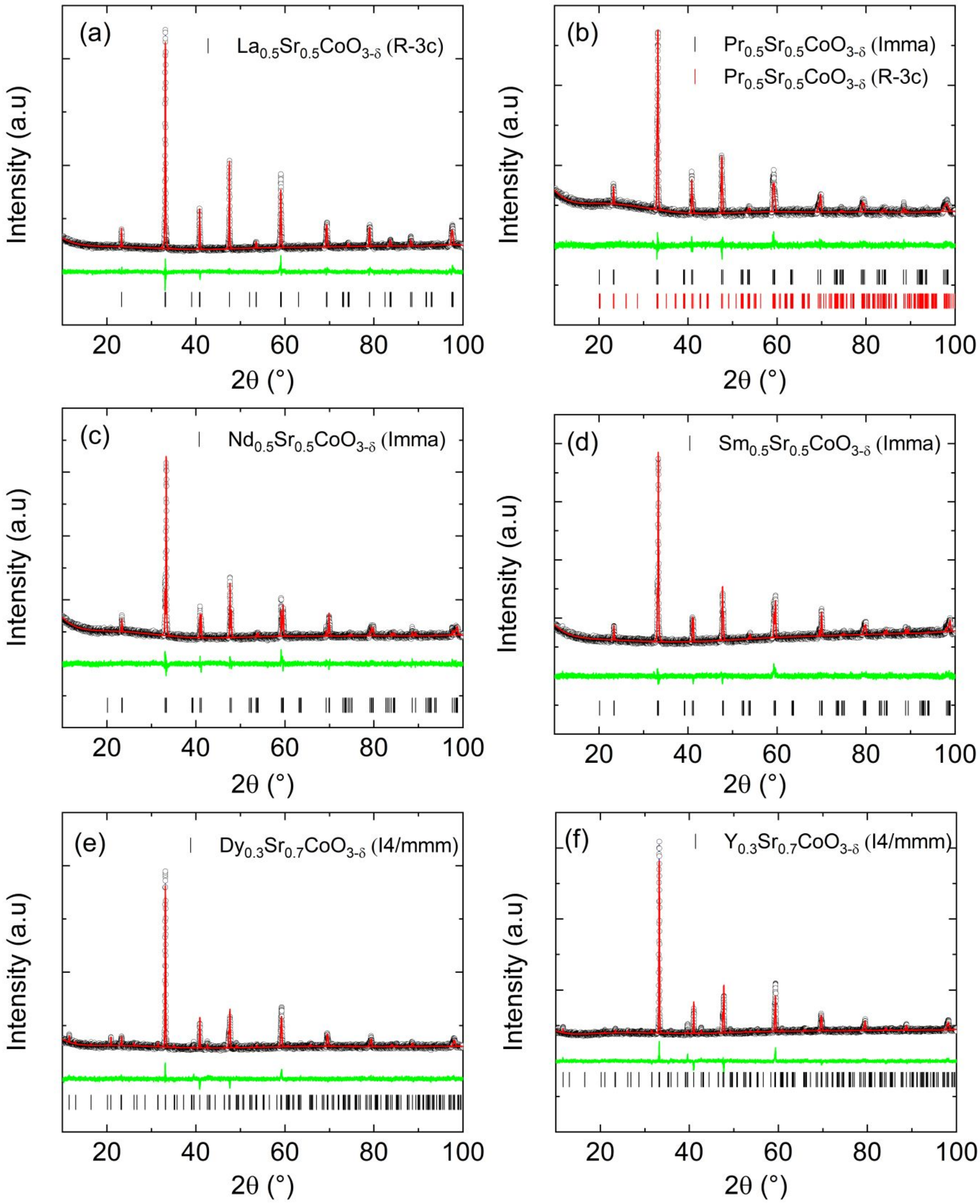

Figure S2 X-ray diffraction pattern (black circles) collected with monochromated Cu Kα radiation, along with the fits (red lines) for (a-f) $Ln_{1-x}Sr_xCoO_{3-\delta}$ (Ln = La, Pr, Nd, Sm, Dy and Y). The difference curves are shown as green lines at the bottom and the Bragg peak positions are indicated by black bars. Minority phase peak positions are displayed by red bars.

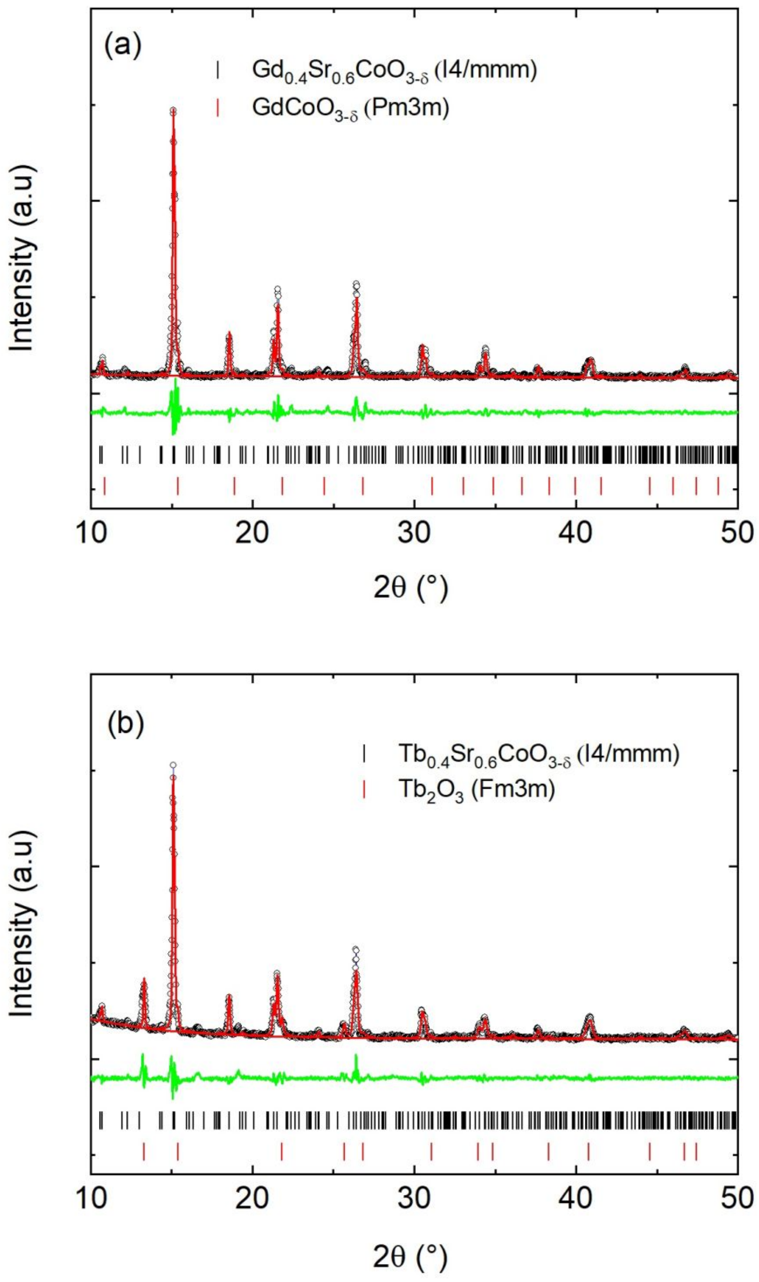

Figure S3 X-ray diffraction pattern (black circles) collected with monochromated Mo Kα radiation, along with the fits (red lines) for (a-b) $Ln_{1-x}Sr_xCoO_{3-\delta}$ (Ln = Gd and Tb). Difference curves are shown as green lines at the bottom and the Bragg peak positions are indicated by black bars. Minority phase peak positions are displayed by red bars.

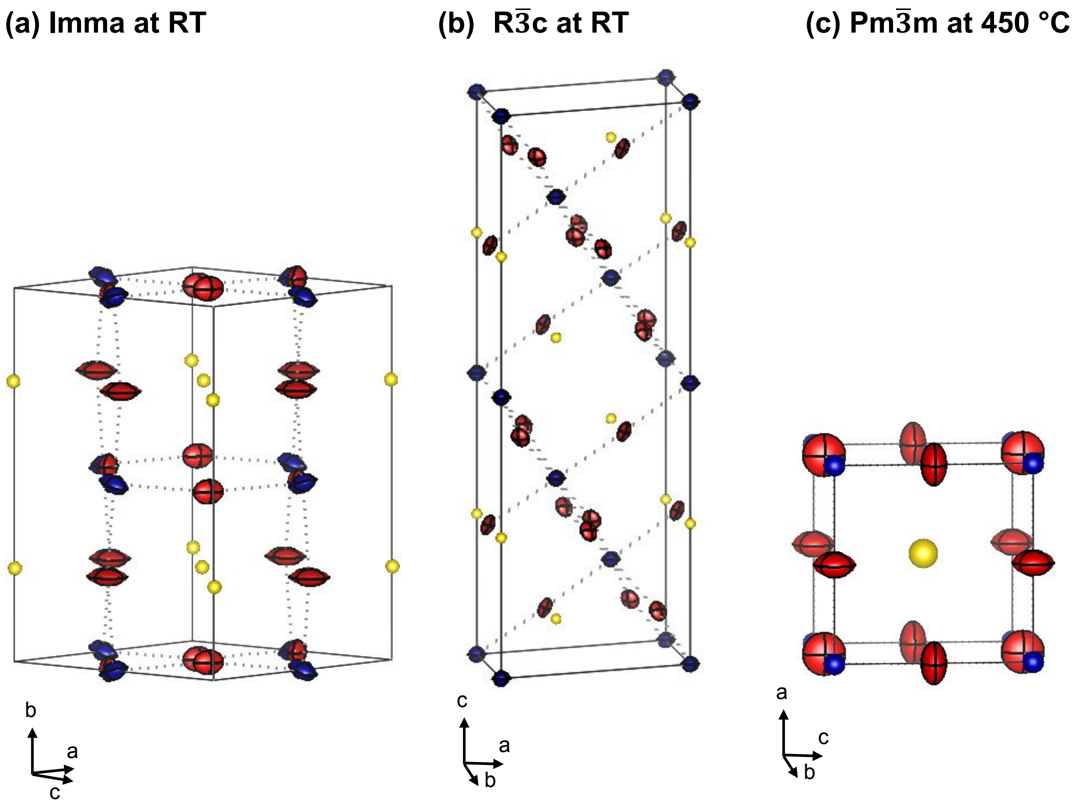

Figure S4. Three dimensional presentations of anisotropic ADPs for $Pr_{0.5}Sr_{0.5}CoO_{3-\delta}$ on heating under flowing N_2. **(a)** √2×2×√2 orthorhombic (Imma) supercell at RT; **(b)** √2×√2×2√3 rhombohedral (R$\bar{3}$c) supercell at RT and **(c)** cubic (Pm$\bar{3}$m) $Pr_{0.5}Sr_{0.5}CoO_{3-\delta}$ structure at 450 °C. ADPs are displayed with thermal ellipsoids (75% probability). Pr/Sr, Co and O atoms are coloured yellow, blue and red.
